# Trefoil Factor 1 Excretion Is Increased in Early Stages of Chronic Kidney Disease

**DOI:** 10.1371/journal.pone.0138312

**Published:** 2015-09-21

**Authors:** Diana Lebherz-Eichinger, Bianca Tudor, Hendrik J. Ankersmit, Thomas Reiter, Martin Haas, Franziska Roth-Walter, Claus G. Krenn, Georg A. Roth

**Affiliations:** 1 Department of Anesthesiology, General Intensive Care and Pain Medicine, Medical University of Vienna, Vienna, Austria; 2 Department of Thoracic Surgery, Medical University of Vienna, Vienna, Austria; 3 Christian Doppler Laboratory for Cardiac and Thoracic Diagnosis and Regeneration, Medical University of Vienna, Vienna, Austria; 4 Division of Nephrology and Dialysis, Department of Medicine III, Medical University of Vienna, Vienna, Austria; 5 Department of Cardiology, University Hospital St. Pölten, St. Pölten, Austria; 6 Comparative Medicine, Messerli Research Institute, University of Veterinary Medicine Vienna, Medical University of Vienna and University of Vienna, Vienna, Austria; 7 RAIC Laboratory 13C1, Medical University of Vienna, Vienna, Austria; The University of Manchester, UNITED KINGDOM

## Abstract

Chronic kidney disease (CKD) is associated with high morbidity and mortality. In many patients CKD is diagnosed late during disease progression. Therefore, the implementation of potential biomarkers may facilitate the early identification of individuals at risk. Trefoil factor family (TFF) peptides promote restitution processes of mucous epithelia and are abundant in the urinary tract. We therefore sought to investigate the TFF peptide levels in patients suffering from CKD and their potential as biomarkers for CKD. We analysed TFF1 and TFF3 in serum and urine of 115 patients with CKD stages 1–5 without dialysis by ELISA. 20 healthy volunteers served as controls. Our results showed, that urinary TFF1 levels were significantly increased with the onset of CKD in stages 1–4 as compared to controls and declined during disease progression (p = 0.003, < 0.001, 0.005, and 0.007. median concentrations: 3.5 pg/mL in controls vs 165.2, 61.1, 17.2, and 15.8 pg/mL in CKD 1–4). TFF1 and TFF3 serum levels were significantly elevated in stages 3–5 as compared to controls (TFF1: p < 0.01; median concentrations: 12.1, 39.7, and 34.5 pg/mL in CKD 3–5. TFF3: p < 0.001; median concentrations: 7.1 ng/mL in controls vs 26.1, 52.8, and 78.8 ng/mL in CKD 3–5). TFF3 excretion was increased in stages 4 and 5 (p < 0.001; median urinary levels: 65.2 ng/mL in controls vs 231.5 and 382.6 ng/mL in CKD 4/5; fractional TFF3 excretion: 6.4 in controls vs 19.6 and 44.1 in CKD 4/5). ROC curve analyses showed, that monitoring TFF peptide levels can predict various CKD stages (AUC urinary/serum TFF > 0.8). In conclusion our results show increased levels of TFF1 and TFF3 in CKD patients with a pronounced elevation of urinary TFF1 in lower CKD stages. Furthermore, TFF1 and TFF3 seems to be differently regulated and show potential to predict various CKD stages, as shown by ROC curve analysis.

## Introduction

Chronic kidney disease (CKD) is associated with high morbidity and mortality and is thus an increasing health problem. Patients suffering from CKD have an elevated risk of cardiovascular diseases and the development of other serious complications [1–5]. CKD is defined either by an estimated glomerular filtration rate (eGFR) of less than 60 mL per minute per 1.73 m^2^ body-surface or by the presence of kidney damage for at least 3 months [2]. Even a minimal decrease in GFR can lead to complications like anaemia or bone disease [2]. Since CKD progression is silent, many patients are identified shortly before the onset of symptomatic renal failure, at a stage where therapeutic options to prevent adverse outcomes are scarce [6]. Therefore, the early detection of individuals at risk is preferable, and can avert progression to total renal failure resulting in kidney replacement therapy by either dialysis or transplantation.

Worsening of kidney function is associated with an increased inflammatory response, apparent in the upregulation of pro-inflammatory cytokines, again triggering the continuous decline of renal function via this vicious circle [7–9]. To prevent further damage and limit cell death renal cells initialize counterreactions, like the initiation of the heat shock response [10–12]. Additionally, the trefoil factor family (TFF) peptides are important proteins involved in the regeneration and repair of the urinary tract. TFF peptides are secretory products of various mucine-producing epithelial cells and promote restitution and regeneration processes of mucous epithelia via induction of cell migration, resistance to proapoptotic stimuli, and angiogenesis [13, 14]. During restitution, mucosal continuity is restored by elongation and migration of epithelial cells to cover denuded areas of damage.

Though TFF peptides have mainy been investigated in the gastrointestinal tract, they were also detected in the urinary tract with TFF3 as the most abundant followed by TFF1 [15]. In preclinical studies TFF3 has already been established as a urinary biomarker for kidney toxicity in animal models [16] and has been successfully shown to be upregulated in CKD patients [17, 18].

To evaluate if TFF peptide levels change during progression of CKD, we investigated TFF1 in serum and urine of 115 patients suffering from CKD stage 1 to 5 in relation to TFF3 concentrations. Furthermore, we calculated fractional TFF1 and TFF3 excretion to detect changes in renal excretion levels independent from glomerular filtration rate.

## Methods

### Patients

The study was approved by the institutional ethics committee of the Medical University of Vienna and was performed in accordance with the Helsinki Declaration of 1975. All participiants have signed informed consent.

We included 115 patients with CKD stage 1 to 5 without dialysis or gastrointestinal diseases and all patients were screened and followed up in the out-patient clinic of the Division of Nephrology and Dialysis, Department of Medicine III, Medical University of Vienna.

CKD was defined as decreased glomerular filtration rate and/or the presence of kidney damage according to the K/DOQI criteria [2]. 20 healthy volunteers served as controls. In the control group kidney diseases, abdominal pain over the previous four weeks, and pregnancy were excluded. The patients’ diagnoses, baseline demographics and laboratory values are shown in Table 1.

**Table 1:** Underlying kidney disease, baseline demographic data and laboratory variables.

	All patients	CKD 1	CKD 2	CKD 3	CKD 4	CKD 5	Controls
N	115	10 (8.8 %)	20 (17.5 %)	40 (35.1 %)	26 (21.9 %)	19 (16.7 %)	20
Age (years)	59 (19-88)	36 (19-61)	50 (19-80)	63 (23-78)	59 (29-88)	65 (20-81)	31 (21-67)
Gender (male/female)	66/48	7/3	8/12	27/13	15/10	9/10	13/7
Kidney disease							
Glomerulonephritis	33	3	8	9	6	7	
Vascular nephropathy	20	2	-	9	8	1	
Diabetic nephropathy	11	1	-	7	3	-	
Polycystic kidney disease	8	2	-	2	2	2	
Hereditary angiomyolipoma	1	-	1	-	-	-	
Interstitial nephropathy	7	-	4	1	1	1	
Urine stasis	6	-	1	1	2	2	
Nephrectomy	4	-	2	-	-	2	
Carcinoma	4	-	1	2	1	-	
Unknown	21	2	3	9	3	4	
Serum creatinine	1.88	0.90	1.01	1.65	2.71	5.00	0.99
(mg dL^-1^)	(0.72-6.88)	(0.72-1.03)	(0.77-1.52)	(1.02-2.34)	(2.04-3.89)	(3.47-6.88)	(0.77-1.30)
Blood urea nitrogen	31.9	12.6	14.3	30.5	49	63.3	13.1
(mg dL^-1^)	(7.1-91.2)	(7.5-17.6)	(7.1-26.2)	(11.6-64.1)	(23.8-91.2)	(31.9-87.3)	(8.2-20)
Urine creatinine	69.9	69.1	74.9	79.4	66.4	41.65	131.7
(mg dL^-1^)	(12.7-294.5)	(22.7-252.9)	(14.8-243.5)	(12.7-294.5)	(29.3-172.1)	(17.7-108.2)	(31.4-316.1)
Urine urea	847	948	1044	931	855	647	
(mg dL^-1^)	(247-2557)	(336-1814)	(247-2557)	(291-2464)	(267-1370)	(273-1381)	
Urine protein (g L^-1^)	0.47	0.06	0.25	0.51	0.55	1.07	
	(< 0.05-6.94)	(< 0.05-0.21)	(< 0.05-4.45)	(< 0.05-2.79)	(<0.05-3.46)	(0.05-6.94)	
CRP (mg dL^-1^)	0.29	0.165	0.14	0.51	0.25	0.56	
	(0.03-8.53)	(0.05-4.31)	(0.05-1.59)	(0.03-3-87)	(0.04-8.53)	(0.05-7.84)	

Data are given as median with range; CKD, chronic kidney disease. Patients specified with carcinoma were suffering from urothelial or renal cell carcinoma and underwent chemotherapy. No renal biopsy was obtained from patients with unknown entity, since proven diagnosis would not have changed treatment planning.

### Laboratory data

A venous blood and urine sample was obtained from all patients and healthy volunteers. In addition, a 24 hour urine sample was collected from a subgroup of 33 patients. Serum and urine samples were centrifuged at 2000 RCF for 10 min at 4°C. Aliquots were transferred into tubes, snap frozen and stored at- 80°C until further use. TFF3 and TFF1 were determined using enzyme-linked immunosorbent assay (ELISA) kits (Human TFF3 Quantikine ELISA Kit and Human TFF1 DuoSet, R&D Systems, Minneapolis, Minnesota, www.rndsystems.com) according to the manufacturer’s instructions. As a substrate, tetramethylbenzidin (TMB; Sigma, St. Louis, Missouri, www.sigmaaldrich.com) was used, and color reaction was stopped with 1% sulfuric acid solution. Optical density was measured with a Victor 3 microplate reader at a wavelength of 450 nm. To overcome methodological deviations serum and urine samples were randomized before analysis.

We performed spike/recovery and linearity testing according to the spike, recovery, and linearity protocol for validating untested samples of R&D systems. For this purpose, known concentrations of trefoil factor peptide were added to the used reagent buffer, urine, and serum samples. Linearity testing was performed by the serial dilution of spiked and unspiked samples. Recovery was obtained by the evaluation of neat and diluted spiked/unspiked samples. Average recovery for TFF1 was 95% for serum samples and 110% for urine samples. Average linearity was 98% for serum samples and 115% for urine samples. Average recovery for TFF3 was 100% for serum samples and 98% for urine samples. Average linearity was 99% for all tested samples. Samples were tested multiple times on different plates and revealed an intra-assay variability of 1% for TFF1 and 2% for TFF3 and an inter-assay variability of 3% for TFF1 and 5% for TFF3, respectively. Testing specificity revealed no significant cross-reactivity between TFF1 and TFF3. The sensitivity was determined by the summation of two standard deviations to the mean optical density of twenty zero samples and by the subsequent calculation of the corresponding concentration. The minimum detectable dose was 3.3 pg/mL for TFF1 and 6.4 pg/mL for TFF3.

The fractional TFF peptide excretion was calculated using the formula: ((urinary TFF peptide x serum creatinine) / (serum TFF peptide x urinary creatinine)) x 100.

### Statistical analysis

TFF protein concentrations in serum and urine as well as fractional excretion were analysed between CKD stage 1 to 5 and the control group. Gaussian distribution was assessed with the D’Agostino-Pearson normality test. Since Gaussian distribution could not be verified for all analyzed groups, the non-parametric Mann-Whitney-test (two-tailed) was used to compare trefoil factor concentrations. According to the Bonferroni adjustment for multiple comparisons, an individual p < 0.01 was necessary to achieve statistical significance at the 5% level.

Correlations between TFF peptide serum and urine levels and several clinical parameters were estimated for all patients and controls by Spearman's correlation coefficient. Unless otherwise specified, data are given as median with range. Statistical analysis was performed using GraphPad Prism Version 5.01 (GraphPad Software, Inc. California, US).

## Results

### Increased total TFF serum and urine levels

**Fig 1:**
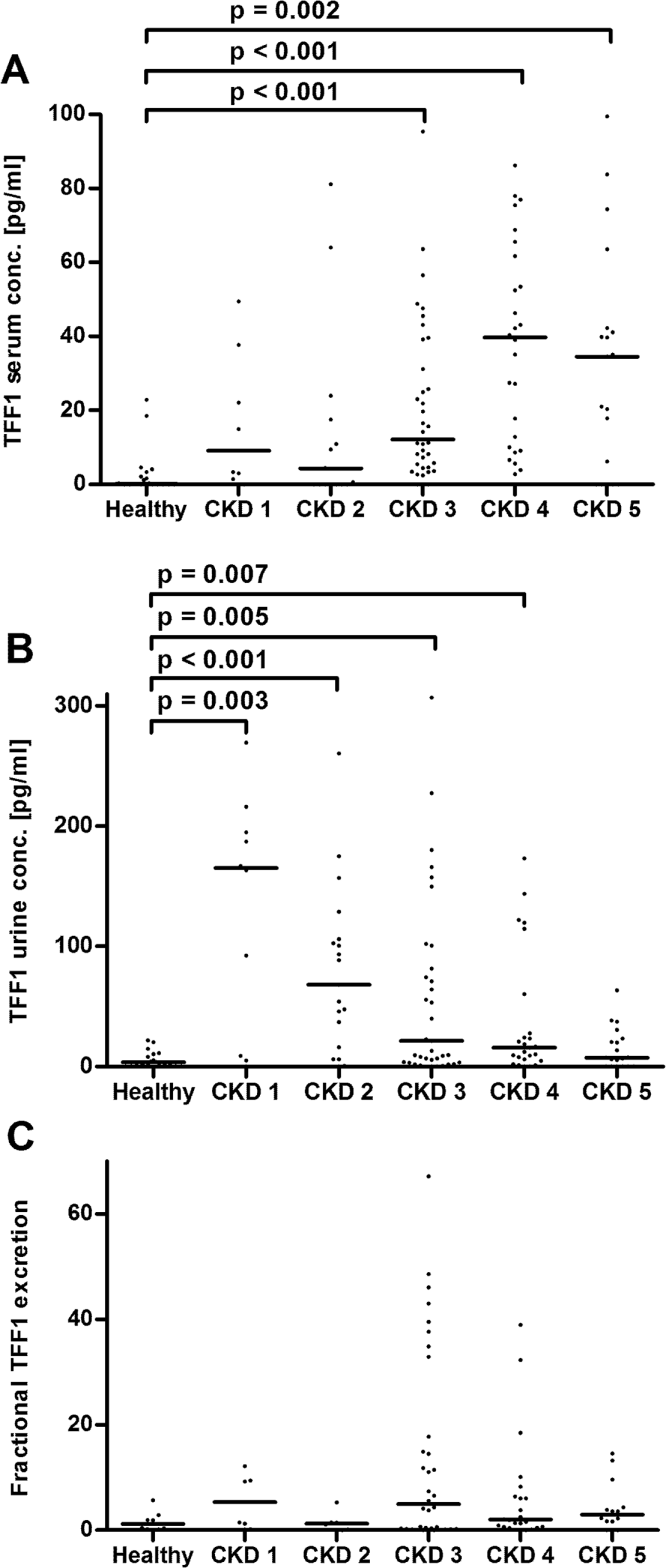
TFF1 levels. Panel A: TFF1 serum concentrations, three data points outside the axis limits in CKD stages 1, 2, and 3. Panel B: Urinary TFF1 levels, three data points outside the axis limits in CKD stage 3. Panel C: Fractional TFF1 excretion, six data points outside the axis limits in controls and CKD stages 1–4. Each dot represents an individual patient. The line indicates the median. CKD, chronic kidney disease. Only significant p-values are given.

**Fig 2:**
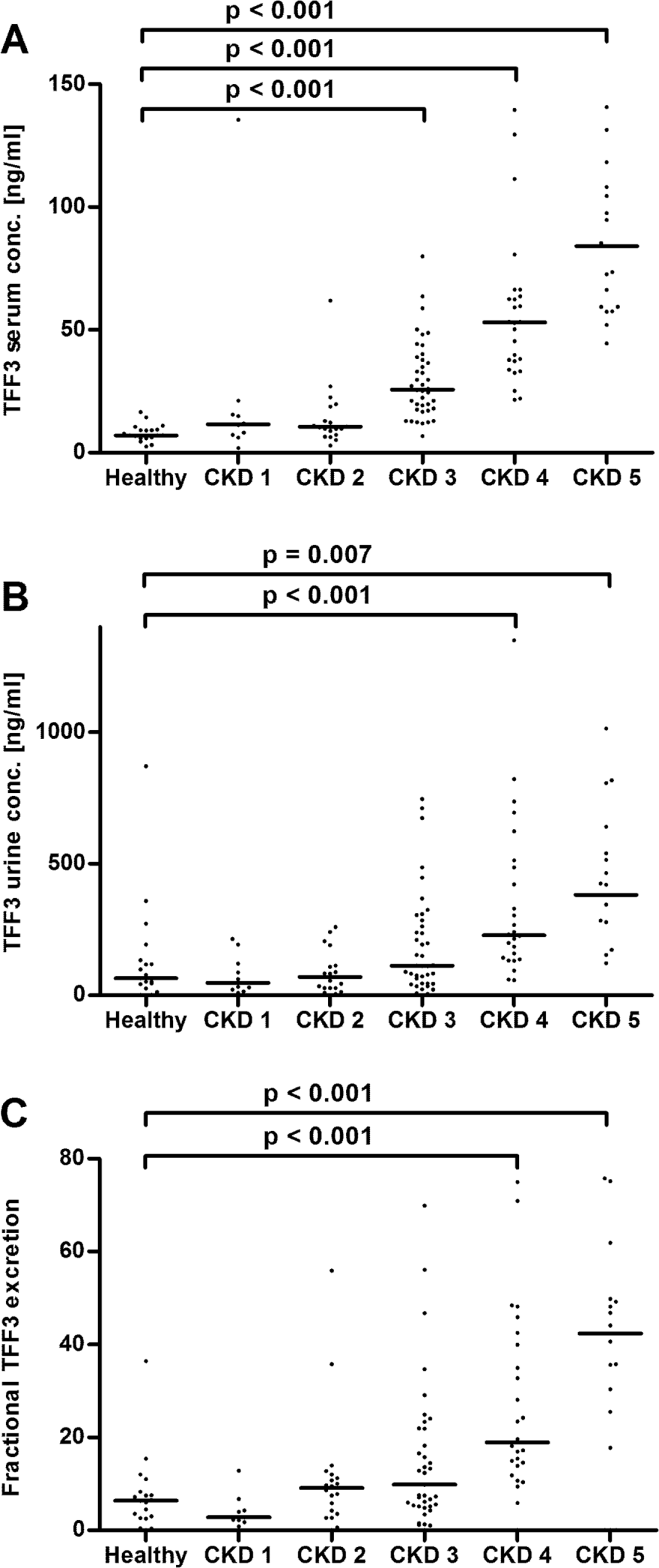
TFF3 levels. Panel A: TFF3 serum levels, two data points outside the axis limits in CKD stages 2 and 5. Panel B: Urinary TFF3 concentrations, one data point outside the axis limits in CKD stage 2. Panel C: Fractional TFF3 excretion, one data point outside the axis limits in CKD stage 5. Each dot represents an individual patient. The line indicates the median. CKD, chronic kidney disease. Only significant p-values are given.

Total TFF1 serum concentration were significantly increased in patients with CKD stages 3 to 5 as compared to the control group (Fig 1A). Total urinary TFF1 levels were significantly higher in CKD stages 1 to 4, as depicted in Fig 1B. Calculation of fractional TFF1 excretion revealed no significant changes (Fig 1C). Total TFF3 serum levels were significantly elevated from stage 3 on, in comparison to the control group (Fig 2A). Total urinary TFF3 concentrations and fractional TFF3 excretion were significantly higher in CKD stages 4 and 5 as compared to controls (Fig 2B and 2C).

### Correlations of total TFF serum and urine levels with clinical and kidney function parameters

The tested parameters were age, C—reactive protein (CRP), eGFR, serum creatinine, urinary albumin, and total urinary protein. In a subset of 33 patients, the correlation between TFF protein levels and the 24h creatinine clearance was evaluated.

There was a strong negative correlation (r <- 0.70) between TFF3 serum levels and creatinine clearance or eGFR (r = - 0.71, p < 0.001 and r = - 0.75, p < 0.001, respectively; Table 2). TFF3 serum levels further show a strong positive correlation to serum creatinine (r = 0.73, p < 0.001) (Table 2). A negative correlation was found between creatinine clearance and urinary TFF3 in the subgroup of patients with a 24-hour urine analysis (r = - 0.55, p = 0.001; Table 2).

**Table 2:** Correlation of TFF serum and urine levels with clinical and kidney function parameters.

	TFF1 serum	TFF1 urine	TFF3 serum	TFF3 urine
Age (115 pairs)	r = 0.44, p < 0.001*	r = - 0.1, p = 0.3	r = 0.35, p < 0.001*	r = 0.17, p = 0.07
Serum creatinine	r = 0.29, p = 0.002*	r = - 0.32, p < 0.001*	r = 0.73, p < 0.001*	r = 0.39, p < 0.001*
(115 pairs)				
Estimated glomerular	r = - 0.32, p < 0.001*	r = 0.32, p < 0.001*	r = - 0.75, p < 0.001*	r = - 0.41, p < 0.001*
filtration rate (115 pairs)				
Total urine protein	r = 0.24, p = 0.01*	r = 0.01, p = 0.9	r = 0.37, p < 0.001*	r = 0.35, p < 0.001*
(114 pairs)				
Urine albumin (88 pairs)	r = 0.35, p < 0.001*	r = 0.02, p = 0.9	r = 0.37, p < 0.001*	r = 0.32, p = 0.003*
Creatinine clearance	r = - 0.2, p = 0.3	r = 0.29, p = 0.1	r = - 0.71, p < 0.001*	r = - 0.55, p = 0.001*
(33 pairs)				
CRP (115 pairs)	r = 0.13, p = 0.2	r = - 0.1, p = 0.3	r = 0.34, p < 0.001*	r = 0.16, p = 0.08

* indicates significance

### ROC curve analysis of total TFF serum and urine levels

**Fig 3:**
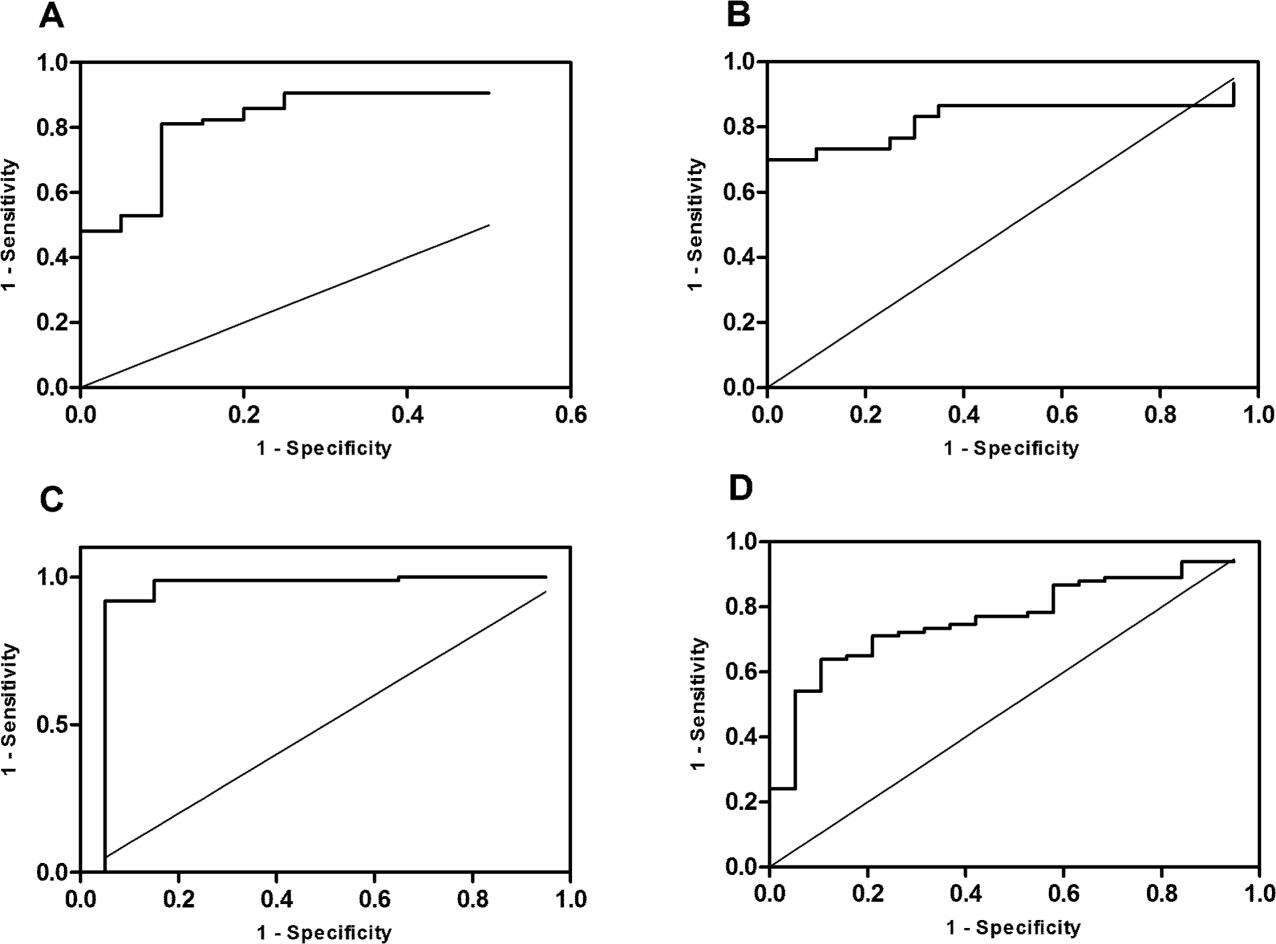
ROC curve analysis. Panel A: ROC curve for serum TFF1 and CKD stage 3 or higher, AUC 0.88 (0.80–0.95, p < 0.001). Panel B: ROC curve for urinary TFF1 and CKD stages 1 and 2, AUC 0.83 (0.71–0.95, p < 0.001). Panel C: ROC curve for serum TFF 3 and CKD stages 3 or higher, AUC 0.94 (0.84–1, p < 0.001). Panel D: ROC curve for fractional TFF3 excretion and CKD stages 3 or higher, AUC 0.77 (0.67–0.87, p < 0.001).

As depicted in Fig 3, ROC curve analysis displayed an area under the curve for the diagnosis of CKD stage 3 or higher of 0.88 (0.80–0.95, p < 0.001) for total serum TFF1 and 0.94 (0.84–1, p < 0.001) for total serum TFF3 concentrations. For fractional TFF3 excretion levels, ROC curve analysis revealed an area under the curve of 0.77 (0.67–0.87, p < 0.001; Fig 3D). Total urine TFF1 concentrations correlated with CKD stage 1 or 2 displayed an area under the curve of 0.83 (0.71–0.95, p < 0.001; Fig 3B).

## Discussion

We were able to demonstrate a pronounced increase in TFF1 urine levels with the onset of CKD. Although the TFF1 urine concentrations abated as kidney function declined, they were still higher than in controls up to CKD stage 4. In contrast, TFF1 serum concentrations constantly increased during progression of chronic renal failure, reaching a significant difference compared to controls in CKD 3 to 5. Furthermore, we found elevated TFF3 peptide excretion levels in patients with CKD 4 and 5, and increased TFF3 serum concentrations from stage 3 on. ROC curve analysis showed the potential of TFF1 and TFF3 to predict various CKD stages.

CKD has a multifactorial origin and is associated with increased cell damage caused by uremic toxins, inflammation, and oxidative stress [19–21]. Persistent inflammation triggers sustained renal damage and contributes to the progression of kidney disease to end-stage renal failure. To minimize cell damage and limit ongoing cell death counterregulatory mechanisms are initialized in order to hold progression of renal dysfunction.

TFF peptides are evolutionarily a highly conserved group of proteins which participate in epithelial protection and restitution. They promote cell migration as well as angiogenesis, limit proapoptotic stimuli, and facilitate leukocyte migration [13, 22]. All TFF peptides are essential for epithelial restitution and can induce cell migration, but they differ in other accessory functions. An example is the tumour suppressive function of TFF1, which has been proven in an animal gastric cancer model [23]. TFF peptides are named after the three-looped structure of their cystein residues, the so called trefoil domain. TFF1 and TFF3 peptides have 60 and 59 amino acids respectively, and contain one trefoil domain each. The highest expression of TFF peptides can be found in the gastrointestinal tract [14]. Moreover, TFF peptides and especially TFF3 have been shown to be secreted by almost all epithelial tissues containing mucus-secreting cells, including the kidney and the urinary tract. Therefore, the involvement of TFF peptides in CKD progression seems likely and has already been proven for TFF3 [17, 18]. To the best of our knowledge, this is the first study evaluating TFF1 serum and urine concentration as well as fractional TFF excretion in patients suffering from CKD.

Our measurements of increased TFF3 serum and urine concentrations are in accordance with the findings from others [17, 18]. Due to ongoing epithelial damage, TFF3 expression is upregulated in chronically inflamed tissues to ensure epithelial restitution and regeneration. TFF peptides are synthesized all along the urinary tract, with TFF3 expression as the most pronounced [15]. Therefore, it is not surprising to find elevated TFF3 levels in patients suffering from CKD, an affliction associated with ongoing renal inflammation and epithelial damage. However, despite increased TFF3 peptide excretion, serum levels remained elevated, indicating continuously increasing TFF3 expression rates to ensure epithelial integrity during CKD progression. On the other hand, sufficient TFF3 excretion might be impeded by diminished filtration due to the cumulative disturbance of the glomerular filtration barrier that accompanies CKD also leading to increased serum levels. However, ROC curve analysis showed the potential of TFF3 serum concentration and fractional TFF3 excretion levels to identify different CKD stages.

Similarly TFF1 is upregulated during inflammation to minimize epithelial damage and maintaining epithelial integrity [13]. Interestingly, we found elevated total TFF1 urine levels with the onset of renal failure. With CKD progression urinary TFF1 levels gradually decreased and returned to concentrations comparable with healthy individuals in CKD 5. In contrast, the elevated TFF1 serum concentrations reached significance only in higher CKD stages, pointing to the kidney as main production site of urinary TFF1 in early CKD. Hence, our findings demonstrate that TFF1 expression seems to be upregulated during the acute phase of kidney disease. Similar results were reported in a rat model of acid induced colitis, in which an elevated TFF1 expression in the distal colon was found during the acute phase of the disease [24]. In contrast, TFF3 expression was downregulated during the acute phase, but increased in the restitution phase [24]. In a methotrexate-induced mucositis model, TFF3 peptides were also minimized during acute phase, but reemerged during regeneration [25]. Those findings are in accordance with our results, showing a continuous increase in TFF3 peptide levels during progression of renal failure to end-stage renal disease. Hence, the initial increase in TFF1 expression and the subsequent secretion of TFF3 peptides in CKD patients might derive from a balanced interplay of TFF peptides trying to ensure mucosal protection during inflammatory and fibrotic processes. However, the observed decrease of total TFF1 urinary concentrations with the concurrent elevation of TFF1 serum levels during disease progression cannot be fully explained by our study. We hypothesize that in the acute phase of renal damage, TFF1 is secreted by the epithelial cells lining the kidney to overcome epithelial damage and is immediately excreted by normal renal clearance. Due to the diminished filtration as well as the increase in angiogenesis in the chronically inflamed kidney, TFF peptide concentration in the blood further increases, which is reflected in the observed rise in TFF1 and TFF3 serum levels at higher CKD stages. However, the initial increase of urinary TFF1 might show the potential of this peptide to early identify individuals at risk and to identify lower CKD stages, as depicted by ROC curve analysis. Furthermore, the combined examination of TFF1 and TFF3 in serum and urine might further improve diagnosis of various CKD stages.

Calculation of fractional TFF clearance revealed that TFF peptide excretion does not seem to depend solely on glomerular filtration. The fractional excretion rates for both proteins above 1, indicate that the source of the TFF peptides are also the epithelial renal cells, which seem to preferentially secrete TFF1 with disease onset followed by TFF3 during disease progression. Due to the decrease of urinary TFF1 in higher CKD stages, the calculation of fractional TFF1 excretion with a formula also including urinary and serum creatinine revealed no differences between patient and healthy probands.

Although this study has produced exciting results, we are aware of some methodological limitations. Even though ELISA testing revealed satisfactory results in recovery, linearity, sensitivity, and coefficient of variations, the kits are not designed for use in clinical testing and thus may remain a source of data bias, possibly influencing results. Moreover, due to the low number of patients not all groups were normally distributed. Consequently, a nonparametric statistical test was applied to detect differences between CKD patients and controls, which might have weakened statistical power. The inclusion of a greater number of patients in early CKD stages may be necessary to evaluate the relevance of TFF1 in the acute phase of kidney injury in future studies. Additionally, this study was planned as a cross-sectional analysis to evaluate TFF peptide levels independent of the underlying renal disease. Our results indicate that TFF1 has the potential to early identify individuals at risk, and that changes in TFF peptide expression might predict disease progression independent of renal affliction. However, we are aware of the limitations of our study by its descriptive design. Finally, the simultaneous evaluation of patients with different causes of CKD might conceal important findings in certain afflictions. Therefore, further clinical testing and longitudinal surveys with more patients are necessary to unravel the role of TFF peptides during progression to end stage renal disease.

## Conclusion

Elevated levels of TFF1 and TFF3 were found in patients suffering from CKD, with TFF1 and TFF3 being differently regulated. Changes in TFF peptide levels might identify aberrations in glomerular filtration and may provide information on disease progression. In particular, the pronounced increase of urinary TFF1 concentration with the onset of CKD indicates that TFF1 might be suitable as a biomarker for the early detection of individuals at risk. However, larger clinical studies and longitudinal surveys are needed to assess the role of TFF peptides in renal failure and their potential as biomarkers.
